# Behavioural intentions to limit social media use among nursing students in Saudi Arabia: a cross-sectional theory of planned behaviour and structural equation modelling study

**DOI:** 10.3389/fpubh.2026.1958042

**Published:** 2026-10-05

**Authors:** Asrar S. Almutairi, Rayhanah R. Almutairi, Abdulaziz M. Alodhailah, Hend N. Almutairi, Waleed M. Alshehri, Mohammed Almutairi

**Affiliations:** 1 Community and Psychiatric Mental Health Nursing Department, College of Nursing, Princess Nourah bint Abdulrahman University, Riyadh, Saudi Arabia; 2 Department of Medical-Surgical Nursing, College of Nursing, King Saud University, Riyadh, Saudi Arabia; 3 School of Archaeology and Anthropology, College of Arts and Social Sciences, The Australian National University, Canberra, ACT, Australia

**Keywords:** attitudes, behavioural intentions, nursing students, perceived behavioural control, Saudi Arabia, social media addiction, subjective norms, theory of planned behaviour

## Abstract

**Background:**

Social media addiction (SMA) carries professional risks for nursing students, yet determinants of their intention to limit social media use are poorly characterised. Using the Theory of Planned Behaviour (TPB), this study examined how attitudes, subjective norms, and perceived behavioural control relate to this intention in Saudi nursing students.

**Methods:**

A cross-sectional survey of 258 nursing students (response rate 91.5%), recruited by stratified random sampling from nursing colleges in Riyadh, Saudi Arabia, was conducted in April–May 2026. Participants completed the Bergen Social Media Addiction Scale (BSMAS) and a 17-item TPB questionnaire. Analyses comprised confirmatory factor analysis (CFA), hierarchical regression, and structural equation modelling (SEM).

**Results:**

In total, 48.4% scored at or above the BSMAS threshold for probable SMA, and perceived behavioural control scored lowest (2.62/5). The four-factor CFA fitted well (CFI = 0.984; RMSEA = 0.029). The final regression model explained 41.2% of variance in intention, with perceived behavioural control the largest standardised predictor (*β* = 0.43), followed by attitudes (*β* = 0.22) and subjective norms (*β* = 0.13). In SEM, perceived behavioural control (*β* = 0.57) and attitudes (*β* = 0.28) were associated with intention; subjective norms were associated with attitudes (*β* = 0.57) but not directly with intention.

**Conclusion:**

Perceived behavioural control was the least strongly held TPB antecedent but the most strongly associated with intention. As the design was cross-sectional and measured only intention, these associations identify candidate targets for nursing education interventions, not causes of behaviour change. Longitudinal studies measuring actual use are needed.

## Introduction

Problematic social media use is a public health concern among university and health professions students. A meta-analysis of 63 samples (34,798 respondents) from 32 nations estimated the prevalence of social media addiction at 5% under strict monothetic classification, 13% under strict polythetic classification, and 25% under more liberal polythetic classification, with higher estimates in collectivist (31%) than individualist (14%) countries (1). In Saudi Arabia, 55.2% of 326 medical students met the BSMAS criterion for social media addiction (2). Estimates therefore vary with the classification method and population studied, but they indicate that problematic use is common among students in the region. As a modifiable, psychosocially determined behaviour, this pattern of use falls within digital health promotion and preventive public health. Nursing students face a dual challenge with social media that sets them apart from students in non-clinical disciplines (3, 4). As young adults, they share the same susceptibility to SMA as any other peer cohort (5, 6). As trainee health professionals, their social media behaviour also carries occupational consequences: lapses in professional boundaries online, inappropriate disclosure of clinical information, and impaired attention during clinical shifts have been associated with problematic social media use in nursing and allied health students (7, 8). The question of what determines nursing students’ intentions to limit potentially addictive social media use is therefore not solely a personal health question but a professional formation one (4, 9).

Behavioural intention is a strong proximal predictor of behaviour across health behaviour theories (10), and the Theory of Planned Behaviour (11) is among the most empirically validated frameworks for predicting such intentions. The TPB identifies three antecedents of intention: attitudes (the individual’s overall evaluative response to performing the behaviour), subjective norms (perceived social pressure from important others to perform or not perform the behaviour), and perceived behavioural control (perceived ease or difficulty of performing the behaviour, equivalent to Bandura’s self-efficacy in many applications). In the SMA reduction context, attitudes capture whether nursing students evaluate limiting their use as personally and professionally beneficial; subjective norms reflect whether peers, faculty, and clinical supervisors are perceived to support such limitation; and perceived behavioural control reflects whether students believe they can actually reduce use despite the structural resistance of platform design (12, 13). Intentions do not always translate into action, however, and the gap between intention and behaviour is substantial across health behaviours (14).

TPB has demonstrated predictive validity across a range of health behaviours, including physical activity adoption, dietary modification, smoking cessation, and alcohol reduction (10, 15). Applications to digital behaviours are more recent. A TPB-based educational intervention has been evaluated for reducing Internet addiction among adolescent girls in southern Iran (13), and a systematic review of school-based programmes for preventing adolescent Internet addiction described a small and heterogeneous prevention evidence base (16). Other work has examined correlates of problematic use rather than intentions; in a Polish sample, for example, Facebook intrusion was associated with fear of missing out and narcissism (17). Few studies have examined intentions to limit social media use through the TPB, and none has done so among nursing students in an Arab cultural context.

The Saudi context is relevant to the TPB’s subjective norms construct. In the present study, subjective norms captured the perceived expectations of peers, faculty, and clinical supervisors. In nursing education, faculty and clinical supervisors are authority figures who convey professional conduct expectations (18–20), and students’ perceptions of supervisor expectations may carry normative weight that does not operate in non-clinical student populations (21, 22). Conversely, if clinical peers model heavy social media use during placement, norms favouring reduction may be weakened. Broader cultural influences, such as family expectations, religious values, and hierarchical authority relationships, may also shape how Saudi students weigh the expectations of others; these influences were not measured in this study and are distinguished here from the subjective norms construct. Testing the TPB in this setting examines whether subjective norms carry the predictive weight the theory assigns them where academic and professional authority are closely linked, with relevance to nursing programmes elsewhere in the Gulf region.

The relationship between SMA severity and TPB constructs matters for intervention design (23). Students at or above the BSMAS probable addiction threshold of 19 (24) may have more entrenched attitudes favouring continued use and weaker intentions to reduce, or they may retain intentions while lacking the perceived control to act on them (25). Which pattern predominates may indicate whether attitudinal or capability-focused interventions are most needed for the highest-risk nursing students.

To our knowledge, no published study has applied the TPB to intentions to limit social media use in a Saudi nursing student population. This study addressed three questions: What attitudes do Saudi nursing students hold toward limiting social media use, and how do subjective norms relate to those attitudes? How do TPB constructs relate to behavioural intentions to reduce social media use? Which constructs are independently associated with intention, and do the TPB measurement and structural models fit the data in this population?

### Significance of the study

This study contributes to evidence on digital wellbeing in nursing education in three ways. First, it provides data from Saudi nursing students, a population in which problematic social media use is common (2) but intentions to limit use have not been examined through the TPB. Second, it tests the factor structure of the TPB measure before estimating the structural model and compares results across regression, SEM, and exploratory subgroup analyses, which allows readers to judge the stability of the associations. Third, identifying which TPB constructs are most strongly associated with intention can inform the design of educational interventions for prospective testing, such as faculty role modelling, teaching on professional digital conduct, and self-regulation skills training. Because the design is cross-sectional, the findings indicate candidate targets rather than causal mechanisms.

## Materials and methods

### Study design and setting

A quantitative cross-sectional survey was conducted among nursing students enrolled in multiple nursing colleges in Riyadh, Saudi Arabia. The study population included undergraduate nursing students from different academic levels, including pre-clinical years (Years 1–2) and clinical-placement years (Years 3–4), as well as postgraduate nursing students in selected specialisations. Multi-institutional recruitment was used to increase sample diversity across nursing education settings in Riyadh. The cross-sectional design was used to assess attitudes, subjective norms, perceived behavioural control, and behavioural intention regarding social media use at a single point in time. The study was reported in accordance with the Strengthening the Reporting of Observational Studies in Epidemiology (STROBE) guidelines for cross-sectional studies (26).

### Participants and sampling

The target population was all enrolled nursing students aged 18 years or older who used at least one social media platform daily. Students enrolled exclusively in distance-learning components and those on academic leave were excluded. Stratified random sampling by academic year and sex ensured proportional representation across year groups. A total of 282 eligible students were invited to participate. Of these, 258 completed usable questionnaires and were included in the final analysis (response rate: 91.5%); the remaining 24 either declined, did not return the questionnaire, or submitted incomplete responses that were excluded prior to analysis. All returned questionnaires were screened for completeness before analysis; because only fully completed questionnaires were retained, no item-level missing data were present in the analysed sample of 258.

Sample size for hierarchical regression was estimated using G*Power 3.1 (seven predictors in the final model, f^2^ = 0.15, alpha = 0.05, power = 0.95), indicating a minimum of 153 participants; the achieved sample of 258 provides power above 0.99 for this effect size. The structural model estimated 39 free parameters, giving approximately 6.6 participants per parameter, which exceeds the ratio of 5:1 often cited as a minimum for maximum likelihood estimation (27).

### Instrumentation

Section 1 collected sociodemographic data including sex, age, academic year, and enrolment level. Section 2 comprised the six-item BSMAS (28), scored from 6 to 30, with scores of 19 or above indicating probable addiction (24); the Arabic version has shown acceptable internal consistency (Cronbach’s alpha = 0.74) (29). Sections 3–6 comprised the researcher-developed TPB questionnaire, constructed following Ajzen’s (11) instrument development guidelines and reviewed by five experts in health psychology and nursing education.

The questionnaire contained 17 TPB items. The Attitudes subscale (6 items; e.g., ‘Reducing my social media use would improve my academic and clinical performance’; Cronbach’s alpha = 0.81) assessed evaluative responses to social media use reduction. The Subjective Norms subscale (4 items; e.g., ‘Clinical supervisors and faculty expect students to use social media responsibly’; alpha = 0.76) captured perceived social pressure from both peer and professional sources. The Perceived Behavioural Control subscale (4 items; e.g., ‘I am confident that I could reduce my social media use during clinical placements if I decided to’; alpha = 0.79) assessed perceived capability. The Behavioural Intention subscale (3 items; e.g., ‘I intend to actively limit my social media use in clinical settings in the next month’; alpha = 0.84) served as the primary outcome. All items were rated on a five-point Likert scale, and each subscale score was calculated as the mean of its items (range 1–5), with higher scores indicating more favourable attitudes, stronger perceived norms, greater perceived control, and stronger intention to limit use. The full item set is available from the corresponding author on request.

### Data collection

Paper-based questionnaires were administered by trained research assistants with written informed consent during lectures, tutorial sessions, and clinical debriefing periods across the participating nursing colleges. Participation was voluntary and anonymous; ethics approval and consent procedures are reported in the Ethics statement. Stratified sampling by academic year and sex was used to reduce selection bias, anonymous self-administration was used to reduce social desirability bias in reporting social media use, and standardised instructions minimised measurement bias arising from differential item interpretation across sites.

### Statistical analysis

IBM SPSS Statistics version 26 was used for descriptive statistics, reliability analysis, correlations, and hierarchical regression, and IBM SPSS AMOS version 26 for confirmatory factor analysis (CFA) and structural equation modelling (SEM). Internal consistency was assessed with Cronbach’s alpha. Before the structural model was interpreted, a four-factor CFA of the 17 TPB items (attitudes, subjective norms, perceived behavioural control, and behavioural intention) was estimated by maximum likelihood.

Model fit was evaluated with the chi-square test, the comparative fit index (CFI), the Tucker–Lewis index (TLI), the root mean square error of approximation (RMSEA) with its 90% confidence interval, and the standardised root mean square residual (SRMR). CFI and TLI values above 0.90 and RMSEA and SRMR values below 0.08 were taken to indicate acceptable fit, with CFI and TLI of 0.95 or above and RMSEA of 0.06 or below indicating good fit (30). Because the chi-square test is sensitive to sample size, it was interpreted alongside the approximate fit indices rather than as the sole criterion. No *post-hoc* covariances were to be added on the basis of modification indices alone.

Pearson correlations examined bivariate associations. Hierarchical regression modelled behavioural intention in three blocks: Block 1 (sex, age, academic year, BSMAS score), Block 2 (attitudes and subjective norms), and Block 3 (perceived behavioural control). Change in R^2^ was assessed at each step. The structural model specified paths from attitudes, subjective norms, and perceived behavioural control to intention and a path from subjective norms to attitudes. The indirect route from subjective norms to intention through attitudes was evaluated with the joint significance test, which requires both component paths to be significant (31).

Two sets of exploratory analyses were conducted. First, six interaction terms (sex and clinical year, each multiplied by attitudes, subjective norms, and perceived behavioural control, using mean-centred predictors) were added simultaneously to the final regression model and evaluated as a block with the F-change test. Second, the final regression model was re-estimated separately for participants at or above the BSMAS threshold (≥ 19) and those below it. Statistical significance was set at *p* < 0.05 (two-tailed).

## Results

### Participant characteristics

Table 1 summarises participant characteristics. The sample was 58.9% female, with a mean age of 23.4 years (SD 4.2), and 125 students (48.4%) scored at or above the BSMAS threshold for probable addiction.

**Table 1:** Participant characteristics (*N* = 258).

Characteristic	n (%)	Mean (SD)
Sex		
Female	152 (58.9)	–
Male	106 (41.1)	–
Age (years)	–	23.4 (4.2)
< 20	48 (18.6)	
20–25	153 (59.3)	
26–30	42 (16.3)	
> 30	15 (5.8)	
Academic year		
Year 1	68 (26.4)	
Year 2	67 (26.0)	
Year 3	63 (24.4)	
Year 4 / Postgraduate	60 (23.3)	
Enrolment level		
Undergraduate	192 (74.4)	
Postgraduate	66 (25.6)	
Daily social media use		
< 1 h	18 (7.0)	
1–3 h	65 (25.2)	
4–6 h	98 (38.0)	
> 6 h	77 (29.8)	
BSMAS total score	**–**	20.3 (5.8)
Probable SMA (≥ 19)	125 (48.4)	–

SMA, social media addiction; BSMAS, Bergen Social Media Addiction Scale. Percentages may not sum to 100 due to rounding.

### Descriptive statistics and reliability

Table 2 presents means, standard deviations, and Cronbach’s alphas for all study instruments. Attitudes were the most favourable TPB construct (mean 3.30, SD 0.72), and perceived behavioural control had the lowest mean (mean 2.62, SD 0.87). Behavioural intention had a mean of 3.10 (SD 0.79); 167 participants (64.7%) scored at or above the scale midpoint of 3. The BSMAS mean was 20.3 (SD 5.8), and 48.4% met the probable addiction criterion. Internal consistency was acceptable for all TPB subscales (alpha = 0.76–0.84).

**Table 2:** Descriptive statistics and reliability for study instruments (*N* = 258).

Variable	Mean	SD	Range	α
Attitudes (6 items)	3.30	0.72	1–5	0.81
Subjective Norms (4 items)	2.87	0.81	1–5	0.76
Perceived Behavioural Control (4 items)	2.62	0.87	1–5	0.79
Behavioural Intention (3 items)	3.10	0.79	1–5	0.84
BSMAS Total Score (6 items)	20.3	5.8	6–30	0.97

SD, standard deviation; α, Cronbach’s alpha; BSMAS, Bergen Social Media Addiction Scale.

### Confirmatory factor analysis

The four-factor measurement model showed good fit: χ^2^(113) = 136.80, *p* = 0.063; CFI = 0.984; TLI = 0.980; RMSEA = 0.029 (90% CI 0.000–0.044); SRMR = 0.043 (Table 3). All 17 items loaded on their intended factors, with standardised loadings ranging from 0.584 to 0.857, supporting the treatment of attitudes, subjective norms, perceived behavioural control, and behavioural intention as distinct constructs in this sample.

**Table 3:** Fit of the confirmatory factor analysis (measurement) model and the structural model (*N* = 258).

Model	χ^2^ (df)	*p*	CFI	TLI	RMSEA (90% CI)	SRMR
Four-factor measurement model (CFA)	136.80 (113)	0.063	0.984	0.980	0.029 (0.000–0.044)	0.043
Structural TPB model (SEM)	173.45 (114)	< 0.001	0.960	0.952	0.045 (0.031–0.058)	0.084

CFI, comparative fit index; TLI, Tucker–Lewis index; RMSEA, root mean square error of approximation; SRMR, standardised root mean square residual. Acceptable fit: CFI and TLI > 0.90; RMSEA and SRMR < 0.08. Standardised CFA loadings ranged from 0.584 to 0.857. No *post-hoc* covariances were added to either model.

### Bivariate correlations

All three TPB constructs were positively and significantly correlated with behavioural intention: perceived behavioural control *r* = 0.58, attitudes *r* = 0.48, and subjective norms *r* = 0.33 (all *p* < 0.001). BSMAS total score was negatively correlated with intention (*r* = −0.28, *p* < 0.001) and with each TPB construct. Attitudes and subjective norms were moderately intercorrelated (*r* = 0.42, *p* < 0.001), consistent with TPB’s prediction of some overlap between evaluative and normative antecedents. The full correlation matrix is presented in Table 4.

**Table 4:** Pearson correlation matrix (*N* = 258).

Variable	1	2	3	4	5
1. Attitudes	–				
2. Subjective Norms	0.42***	–			
3. Perceived Behavioural Control	0.45***	0.23***	–		
4. Behavioural Intention	0.48***	0.33***	0.58***	–	
5. BSMAS Total	−0.23***	−0.21***	−0.35***	−0.28***	–

***p < 0.001. BSMAS, Bergen Social Media Addiction Scale.

### Hierarchical regression

Table 5 presents the hierarchical regression results. Block 1 (demographics and BSMAS score) explained 7.8% of the variance in intention (R^2^ = 0.078; *F*(4, 253) = 5.35, *p* < 0.001), with BSMAS score the only significant term (*β* = −0.28, *p* < 0.001). Block 2 (attitudes and subjective norms) increased this to 27.6% (ΔR^2^ = 0.198, *p* < 0.001). Block 3 (perceived behavioural control) produced a final R^2^ of 0.412 (adjusted R^2^ = 0.395; ΔR^2^ = 0.136, *p* < 0.001). In the final model, perceived behavioural control had the largest standardised coefficient (*β* = 0.433, *p* < 0.001), followed by attitudes (*β* = 0.220, *p* < 0.001) and subjective norms (*β* = 0.129, *p* = 0.018). BSMAS score was no longer significant once perceived behavioural control was entered (*β* = −0.051, *p* = 0.335). Sex, academic year, and age were not associated with intention.

**Table 5:** Hierarchical regression predicting behavioural intention to reduce social media use among nursing students (*N* = 258).

Predictor	B	SE	β	*p*	95% CI
Block 1 (R^2^ = 0.078; *F*(4, 253) = 5.35, *p* < 0.001)					
Female sex (ref: male)	0.031	0.097	0.020	0.745	−0.159, 0.222
Clinical year (ref: pre-clinical)	−0.030	0.095	−0.019	0.754	−0.218, 0.158
Age (years)	0.000	0.011	0.002	0.980	−0.022, 0.023
BSMAS total score	−0.038	0.008	−0.281***	< 0.001	−0.054, −0.022
Block 2 (ΔR^2^ = 0.198, *p* < 0.001; cumulative R^2^ = 0.276)					
Attitudes	0.420	0.065	0.387***	< 0.001	0.291, 0.549
Subjective norms	0.134	0.058	0.137*	0.023	0.019, 0.248
Block 3 (ΔR^2^ = 0.136, *p* < 0.001; final R^2^ = 0.412; adjusted R^2^ = 0.395)					
Attitudes	0.239	0.064	0.220***	< 0.001	0.113, 0.364
Subjective norms	0.125	0.053	0.129*	0.018	0.022, 0.229
Perceived behavioural control	0.390	0.051	0.433***	< 0.001	0.289, 0.491
BSMAS total score	−0.007	0.007	−0.051	0.335	−0.021, 0.007

* *p* < 0.05; *** *p* < 0.001. B, unstandardised coefficient; SE, standard error; β, standardised coefficient; CI, confidence interval; BSMAS, Bergen Social Media Addiction Scale. Block 1 and Block 2 coefficients are shown at entry; Block 3 coefficients are from the final model, which also included sex, clinical year, and age.

### Exploratory interaction and sensitivity analyses

Adding the six sex × TPB and clinical-year × TPB interaction terms did not improve the final regression model as a block (ΔR^2^ = 0.018, F-change(6, 244) = 1.30, *p* = 0.260). One term, clinical year × subjective norms, had a nominal *p* value of 0.030 (B = 0.234; Holm-adjusted *p* = 0.182); it was not interpreted as a reliable moderation effect because the block was non-significant and six terms were tested. In the BSMAS sensitivity analyses (Table 6), perceived behavioural control remained the largest standardised predictor of intention among participants with probable SMA (*n* = 125; *β* = 0.429, *p* < 0.001) and among those below the threshold (*n* = 133; *β* = 0.412, *p* < 0.001).

**Table 6:** Sensitivity analysis: final regression model re-estimated by BSMAS threshold group.

Sample	n	ATT β	SN β	PBC β	BSMAS β	R^2^
Full sample	258	0.220***	0.129*	0.433***	−0.051	0.412
BSMAS ≥ 19 (probable SMA)	125	0.259**	0.085	0.429***	−0.032	0.381
BSMAS < 19	133	0.178*	0.185*	0.412***	−0.093	0.396

*p < 0.05; ** *p* < 0.01; *** *p* < 0.001. Standardised coefficients from the full Block 3 model, which also included sex, clinical year, and age. ATT, attitudes; SN, subjective norms; PBC, perceived behavioural control; BSMAS, Bergen Social Media Addiction Scale; SMA, social media addiction.

### Structural equation model

The structural model showed acceptable fit on CFI, TLI, and RMSEA: χ^2^(114) = 173.45, *p* < 0.001; CFI = 0.960; TLI = 0.952; RMSEA = 0.045 (90% CI 0.031–0.058). SRMR was 0.084, slightly above the 0.08 criterion (Table 3). No *post-hoc* covariances were added to improve fit. Figure 1 shows the standardised latent paths. Perceived behavioural control had the largest path to intention (*β* = 0.566, *p* < 0.001), followed by attitudes (*β* = 0.284, *p* = 0.001). Subjective norms were strongly associated with attitudes (*β* = 0.569, *p* < 0.001), but the direct path from subjective norms to intention was not significant (*β* = 0.076, *p* = 0.367). Both component paths of the indirect route from subjective norms to intention through attitudes were significant, and the product of the two standardised paths was 0.16.

**Figure 1:**
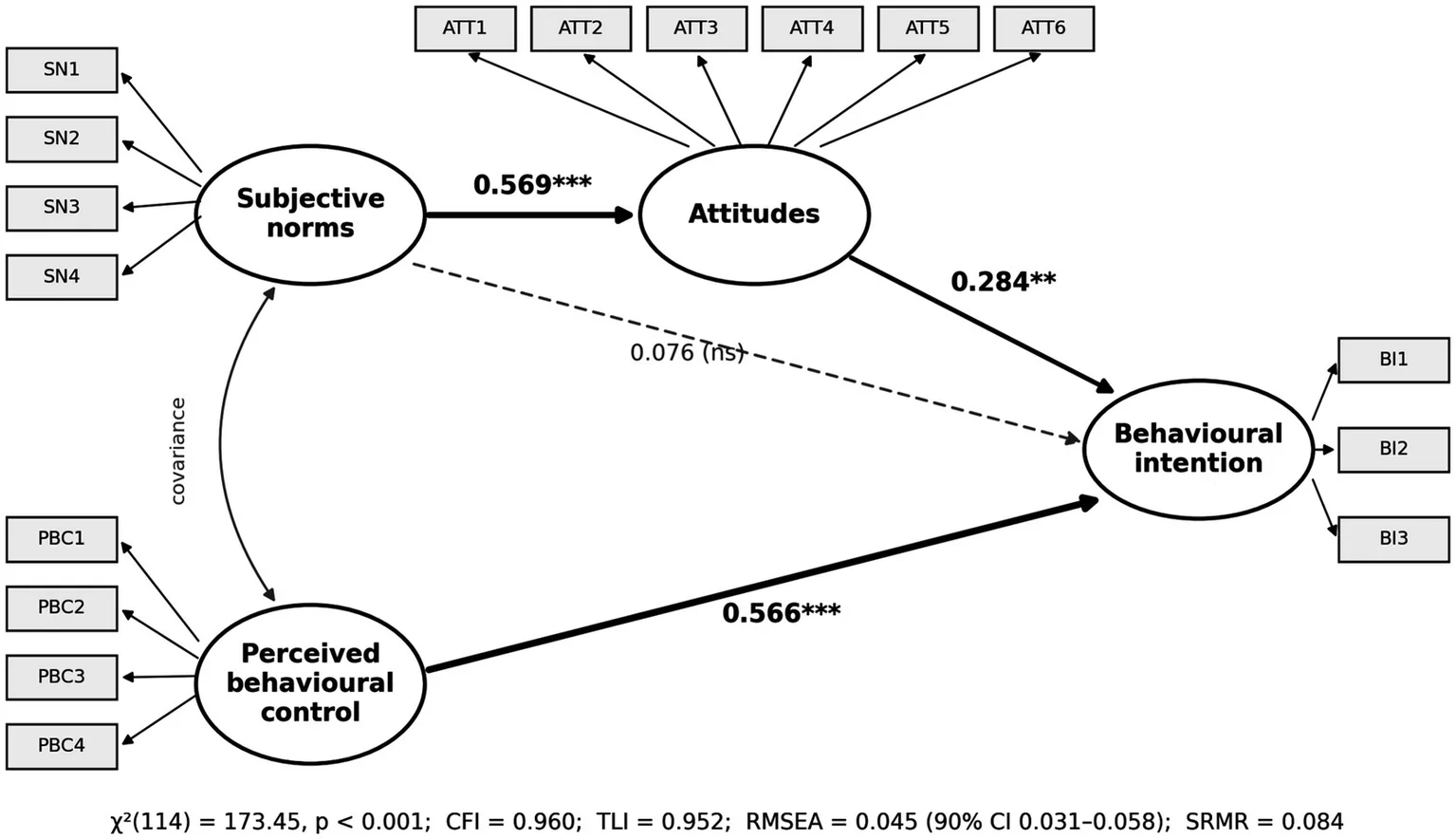
Structural equation model of theory of planned behaviour constructs and behavioural intention to limit social media use (*N* = 258). Standardised coefficients are shown. Ellipses represent latent variables and rectangles observed items; solid arrows indicate significant paths, the dashed arrow a non-significant path, and the curved double-headed arrow the estimated covariance between subjective norms and perceived behavioural control. ns, not significant; ** *p* < 0.01; *** *p* < 0.001.

## Discussion

In this sample of Saudi nursing students, the TPB constructs, together with demographic characteristics and BSMAS score, accounted for 41.2% of the variance in intention to limit social media use in the final regression model, which is comparable to the proportion of variance in intention reported across health behaviours in a meta-analysis of prospective TPB studies (15). The measurement model fitted well, supporting the use of the four TPB subscales as distinct constructs in this population.

The regression and SEM analyses gave a consistent picture. Perceived behavioural control had the largest standardised coefficient in the final regression model (*β* = 0.433) and the largest structural path in SEM (*β* = 0.566), and attitudes were significant in both analyses. Subjective norms differed between the two approaches: they were a small but significant predictor in the observed-score regression (*β* = 0.129) but had no significant direct latent path to intention in SEM (*β* = 0.076), whereas their path to attitudes was strong (*β* = 0.569). The two analyses estimate different quantities. Regression uses observed scale means, which contain measurement error, and estimates the unique contribution of each predictor after adjustment for the others; SEM models the constructs as latent variables and estimates all paths simultaneously, including the path from subjective norms to attitudes. The pattern is compatible with an indirect normative pathway through attitudes, but cross-sectional data cannot establish that norms shape attitudes rather than the reverse, or that both reflect a shared influence.

Perceived behavioural control had the lowest mean of the TPB antecedents (2.62/5), yet it had the largest association with intention in both analyses. This combination is relevant for nursing faculty: students who express reasonably favourable attitudes toward limiting use but lack confidence in their ability to reduce use may translate intention into action less reliably (32–34). Platform architecture, which incorporates algorithmic recommendation, infinite scrolling, notification engineering, and streak-based reward systems, is designed to sustain engagement and may make self-regulation harder (35, 36). Nursing students’ low perceived behavioural control may therefore reflect this environment rather than an underestimation of personal capability (37). Curricula that teach digital self-regulation as a professional competency, including setting use boundaries during clinical shifts, disabling non-essential notifications, and using screen-time management features, are candidate approaches for strengthening perceived behavioural control, although their effects on intention and behaviour need to be tested prospectively.

The strong association between subjective norms and attitudes, alongside the non-significant direct path from norms to intention, has relevance for nursing education. Faculty and clinical supervisors convey professional expectations (18, 19, 21), and students who perceived that these figures expected responsible social media use also held more favourable attitudes toward limiting use. One interpretation is that normative expectations become internalised as personal evaluations; another is that students with favourable attitudes perceive their supervisors’ expectations more readily. The present data cannot distinguish these explanations. If the first is correct, faculty role modelling would operate partly through students’ attitudes rather than through normative pressure alone, a hypothesis that longitudinal or intervention studies could test. Family influence, religious values, and hierarchical authority outside the educational setting may also contribute to these associations but were not measured here.

The low subjective norms score (mean 2.87/5) indicates that nursing students did not strongly perceive peer or professional support for limiting social media use. This is notable given the professional conduct expectations that exist within nursing education. It may indicate that professional expectations around digital conduct are not being communicated clearly, or that peer social media culture overshadows perceptions of institutional norms. Al Kuraydis et al. (38) reported an association between social media addiction and social phobia among Saudi adolescents; in a nursing student context, where social media is also used for peer academic collaboration and information sharing, concerns about social exclusion may make limiting use harder. Messages that present responsible digital conduct as a professional nursing value, rather than a personal restriction, could be evaluated as a way to shift this perception.

BSMAS score was negatively associated with intention at entry (*β* = −0.28) but not in the final model once perceived behavioural control was added (*β* = −0.05, *p* = 0.335). In cross-sectional data, this pattern indicates that the negative association between addiction severity and intention overlaps with lower perceived control; it does not show that perceived control explains the relationship. The sensitivity analyses showed a similar pattern of predictors above and below the BSMAS threshold, with perceived behavioural control the largest predictor in both groups. Nearly half of this sample (48.4%) met the probable addiction criterion, and this group may need support beyond cohort-wide education. Confidential wellbeing screening, for example with the BSMAS during clinical orientation, combined with locally approved stepped referral to counselling or student support services, is one option that nursing colleges could evaluate.

Neither sex nor academic year was associated with intention, and the interaction terms involving these variables did not improve the model as a block. The single nominally significant clinical year × subjective norms term should be regarded as a hypothesis for studies powered to test moderation, not as evidence that normative influences differ between pre-clinical and clinical students.

For nursing education, the findings suggest components that could be combined and tested in a prospective evaluation rather than a single awareness campaign. Candidate components include case discussions of professional digital conduct drawn from clinical practice; explicit modelling of responsible device use by faculty and clinical supervisors; guided reflection on the academic, clinical, and professional consequences of social media use; and skills training in notification management, screen-time planning, and scheduled device-free periods during clinical placements. These components correspond to the three TPB antecedents, but the present cross-sectional findings do not show that any of them would change intention or behaviour. Because nursing programmes elsewhere in the Gulf share features of the Saudi educational context, this approach may be relevant beyond Riyadh, pending replication.

### Strengths and limitations

Strengths of this study include the nursing-specific sample, stratified sampling with a high response rate, testing of the TPB measurement model by CFA before the structural model was interpreted, and the use of regression, SEM, interaction, and sensitivity analyses to examine the stability of the findings. Several limitations apply. The cross-sectional design prevents conclusions about temporal order or causation; the associations reported here, including the indirect pathway from subjective norms through attitudes, may reflect reverse or shared influences. Only behavioural intention was measured. Intentions do not always translate into action, and changes in intention are typically followed by smaller changes in behaviour (14); future longitudinal studies should measure actual social media use, ideally with device-recorded screen time. Although CFA supported the four-factor structure of the researcher-developed TPB instrument in this sample, the structure should be replicated in independent samples, and the SRMR of the structural model was slightly above the conventional criterion. Self-reported measures may be subject to social desirability bias, particularly among students aware of professional expectations. Recruitment from nursing colleges in Riyadh may limit generalisability to nursing students in other regions of Saudi Arabia. Finally, the interaction and sensitivity analyses were exploratory and not powered to detect small moderation effects, and broader cultural influences such as family expectations and religious values were not measured.

## Conclusion

In this cross-sectional study, TPB constructs were associated with Saudi nursing students’ intentions to limit social media use, and the four-factor TPB measure fitted the data well. Perceived behavioural control was the least strongly held antecedent but had the strongest association with intention in both regression and SEM, and subjective norms were associated with intention mainly through their association with attitudes. These findings identify perceived behavioural control, attitudes, and the professional expectations conveyed by faculty and clinical supervisors as candidate targets for nursing education interventions. Whether such interventions change intention and actual social media use needs to be tested in longitudinal and intervention studies. Given that 48.4% of participants met the criterion for probable addiction, confidential screening with stepped referral merits evaluation alongside any educational programme.

## Data Availability

The raw data supporting the conclusions of this article will be made available by the authors, without undue reservation.
