# Supplementary material for: Behavioural intentions to limit social media use among nursing students in Saudi Arabia: a cross-sectional theory of planned behaviour and structural equation modelling study
Source: Front Public Health. 2026 Oct 5;14:1958042. doi: 10.3389/fpubh.2026.1958042 (PMC13677849; doi:10.3389/fpubh.2026.1958042)
Supplement: Supplementary file 1 [file Table_1.DOCX]

# **STROBE Statement — Checklist for Cross-Sectional Studies**

| **Item** | **STROBE recommendation** | **Reported on page/section** |
| --- | --- | --- |
| 1a | Indicate the study's design with a commonly used term in the title or abstract | Title, p.1 (“...A Cross-Sectional Theory of Planned Behaviour and Structural Equation Modelling Study”); Abstract, p.1 |
| 1b | Provide an informative and balanced summary of what was done and found | Abstract, p.1 (structured Background/Methods/Results/Conclusions) |
| 2 | Explain the scientific background and rationale for the investigation | Introduction, pp.2–5 |
| 3 | State specific objectives, including any prespecified hypotheses | Introduction, pp.4–5 (H1–H4; three research questions) |
| 4 | Present key elements of study design early in the paper | Methods – Study Design and Setting, p.5 |
| 5 | Describe the setting, locations, and relevant dates | Methods – Study Design and Setting, p.5 (Riyadh; April–May 2026) |
| 6a | Give eligibility criteria, sources and methods of participant selection | Methods – Participants and Sampling, p.6 |
| 7 | Clearly define all outcomes, exposures, predictors, confounders, effect modifiers | Methods – Instrumentation, p.6 |
| 8 | For each variable, give sources of data and details of measurement | Methods – Instrumentation, p.6 (BSMAS, TPB subscales, alphas) |
| 9 | Describe any efforts to address potential sources of bias | Methods – Data Collection and Ethics, p.7 |
| 10 | Explain how the study size was arrived at | Methods – Participants and Sampling, p.6 (G*Power; SEM 10:1 ratio) |
| 11 | Explain how quantitative variables were handled in the analyses | Methods – Statistical Analysis, pp.7–8 |
| 12a | Describe all statistical methods, including those to control for confounding | Methods – Statistical Analysis, pp.7–8 |
| 12b | Describe any methods used to examine subgroups and interactions | Methods – Statistical Analysis, p.8; Strengths and Limitations, p.16 |
| 12c | Explain how missing data were addressed | Methods – Participants and Sampling, p.6 |
| 12d | If applicable, describe analytical methods taking sampling strategy into account | Methods – Participants and Sampling, p.6 (stratified sampling; 91.5% response) |
| 12e | Describe any sensitivity analyses | Methods – Statistical Analysis, p.8; Strengths and Limitations, p.16 |
| 13a | Report numbers at each stage of the study | Methods – Participants and Sampling, p.6 (282 invited → 258 analysed, 24 excluded; response rate 91.5%) |
| 13b | Give reasons for non-participation at each stage | Methods – Participants and Sampling, p.6 (declined, not returned, or incomplete) |
| 13c | Consider use of a flow diagram | Not present as a figure |
| 14a | Give characteristics of study participants and information on exposures/confounders | Table 1, p.8 (sex, age, academic year, enrolment level, daily social media use, BSMAS caseness; N = 258) |
| 14b | Indicate number of participants with missing data for each variable | Methods – Participants and Sampling, p.6 |
| 15 | Report outcome data | Table 2, p.9; Results narrative, p.9 |
| 16a | Give unadjusted and adjusted estimates and their precision (e.g., 95% CI) | Table 4, pp.11–12; SEM paths with CIs, p.12 |
| 16b | Report category boundaries when continuous variables were categorized | Methods – Instrumentation, p.6 (BSMAS threshold ≥19); Table 1, p.8 (age/use categories) |
| 16c | Consider translating relative risk into absolute risk for a meaningful time period | Not applicable |
| 17 | Report other analyses done (e.g., subgroup, sensitivity) | Strengths and Limitations, p.16 |
| 18 | Summarise key results with reference to study objectives | Discussion, opening paragraph, p.13 |
| 19 | Discuss limitations, direction and magnitude of any bias | Strengths and Limitations, p.16 |
| 20 | Give a cautious overall interpretation considering objectives, limitations, multiplicity, similar studies | Discussion, pp.13–15 |
| 21 | Discuss the generalisability (external validity) of the study results | Strengths and Limitations, p.16; international-contribution paragraph, Introduction p.4 |
| 22 | Give the source of funding and the role of the funders | Declarations – Funding, p.17 (Ongoing Research Funding Program, ORFFT-2026-928, KSU) |
